# Integrating External and Internal Load for Monitoring Fitness and Fatigue Status in Standard Microcycles in Elite Rink Hockey

**DOI:** 10.3389/fphys.2021.698463

**Published:** 2021-06-29

**Authors:** Daniel Fernández, David Moya, Joan A. Cadefau, Gerard Carmona

**Affiliations:** ^1^Sports Performance Area, Futbol Club Barcelona, Barcelona, Spain; ^2^Barça Innovation Hub, Futbol Club Barcelona, Barcelona, Spain; ^3^Institut Nacional d'Educació Física de Catalunya (INEFC), Universitat de Barcelona (UB), Barcelona, Spain; ^4^Escola Superior de Ciències de la Salut, Universitat Pompeu Fabra (UPF), Mataró, Spain

**Keywords:** team sport, load control, ultrawide-band, LPS, GPS

## Abstract

The aims of this study were 3-fold: firstly, to present an integrative approach to external and internal load dynamics for monitoring fitness and fatigue status of specific in-court rink hockey training sessions in a standard microcycle; secondly, to assess the differences between training sessions and matches; the third and final aim was to assess the association between external and internal load metrics. The external load, using a local positioning system, and internal load, using the declared rate of perceived exertion, were measured during 23 in-season microcycles for nine top-level players. Training load data were analysed with regard to the number of days before or after a match [match day (MD) minus or plus]. In relation to the first aim, internal and external load metrics merged into a single integrated system using pooled data *z*-scores provided an invisible monitoring tool that places the players in the fitness-fatigue continuum throughout the different microcycle sessions. In this regard, MD-4 and MD-1 sessions tend to place, with a low dispersion, the players in a “low external and internal load” zone. On the contrary, in MD-3 and MD-2 sessions, as well as in MD, in which higher loads were recorded, most of the players were within a “high external and internal load” zone with a tendency towards dispersion towards the fitness or fatigue zones. Finally, and with regard to the second and third aims, an inverted “*U*-shape” load dynamic related to the specific goals of each training session was the main finding in terms of comparison between MD; a load peak between MD-3 and MD-2 sessions and a significant decrease in all the load variables in MD-1 sessions were found; and high-to-low correlations were found between external and internal load metrics. This study presents an integrative approach to the external and internal load of players for monitoring fitness and fatigue status during a standard microcycle in rink hockey that might provide team sport staff members with a deeper understanding of load distribution in the microcycle in relation to the match.

## Introduction

Workload in the context of sports training has been defined as the input variable which, assuming a certain level of stress, is manipulated to obtain a desired response (Impellizzeri et al., 2019). While training load is totally manipulable, competition load, due to its intrinsic characteristics such as results, place in the league table, stage of the season or type of league, is much less so and is limited to the possibility of regulating player exposure to it (minutes of play). Load can be described as internal or external (Impellizzeri et al., 2019). External load (EL) is the work completed by the player independently of his or her internal characteristics; for example, it is described in terms of distance, accelerations, decelerations, or sprints, among others (Varley and Aughey, 2013). The resulting physiological, psychological, and biomechanical stress imposed, described as internal load (IL), drives player adaptation response (Vanrenterghem et al., 2017). The outcome of any training intervention is therefore the consequence of both EL and IL, hence reliable monitoring tools are crucial to the optimisation of athletic performance (Impellizzeri et al., 2019) and to a better understanding of the factors affecting sport performance and recovery, since the uncoupling or divergence of EL and IL may differentiate between a non—fatigued and a fatigued athlete (Thorpe et al., 2017). Current models of the fitness—fatigue relationship use the association between EL and IL (Delaney et al., 2018), considering “fatigue” as the ability to complete a task or training session with an altered IL response which recent had been achievable with a lower IL response and “fitness state” as the opposite, the ability to accumulate a given EL with a lower IL response.

Thanks to tracking technology, athletes, coaches and sport scientists, can easily compile EL parameters to help practitioners and coaches to plan, evaluate, structure and optimise their training methodology (Borresen and Lambert, 2009). The use of electronic performance and tracking systems has helped to compare competition demands to training session drills and consequently modulate the intensity and volume of these training sessions according to the match day (Buchheit et al., 2014). Moreover, the use of subjective scales to record the declared exertion also makes it possible to obtain information about IL (Foster et al., 2001).

The organisation of training loads can be divided into macrocycles, mesocycles and microcycles (Naclerio et al., 2013). These divisions make it possible to organise the load from very large time cycles (for example macrocycles that encompass two seasons), through mesocycles that group several weeks or months of training, up to weekly microcycles. The most recent research in team sports is beginning to identify the microcycle as the most important planning unit in this type of sport in this specific case, understanding the microcycle not as the training week, but as the time that passes between one game and another (Tarragó et al., 2019). Indeed, many studies have provided comprehensive information about EL of outdoor elite team sports during matches, and some studies have furnished the same information about indoor elite team sports (Vázquez-Guerrero et al., 2019; Gómez-Carmona et al., 2020; Ribeiro et al., 2020), including high-intensity actions and metabolic variables. Research relating elite teams' in-season microcycles in outdoor sports is on the increase (Akenhead et al., 2016; Martín-García et al., 2018; Oliveira et al., 2019) but is virtually non-existent in indoor sports (Illa et al., 2020).

Being an indoor sport, rink hockey is a team sport involving skates and a stick and is characterised by high-intensity actions such as accelerations, decelerations, sprints, and changes of direction (Fernández et al., 2020b). Rink hockey is played in a 40 × 20 m court like futsal or handball, but with fences, a structural difference that means that there are fewer interruptions than in these other sports. It is played by two teams of four players and a goalkeeper, and official matches have two halves of 25 min; the number of players and the court size make rink hockey more similar to futsal than to basketball and handball in terms of density of players. Finally, the use of roller skates makes the movement of the players on the court much easier; they are able to cover large distances, sometimes without effort. Finally, decelerations and accelerations are the key actions in this sport (Fernández et al., 2020b). While the use of technology in certain team sports modalities has increased in the last decade, research into conditional demands is scant. Some research has addressed official matches (Merino Tantiña et al., 2014; Fernández et al., 2020b) and the most demanding passages in certain drills (Fernández et al., 2020a), although microcycle training load dynamics has never been described.

Accordingly, this research pursued three aims: (i) to present an integrative approach to EL and IL dynamics for monitoring the fitness and fatigue status in the specific in-court training sessions of an elite rink hockey team for a standard one-match-per-week microcycle; (ii) to evaluate the differences in EL and IL metrics between training sessions and match; and (iii) to assess the association between EL and IL metrics.

## Materials and Methods

### Participants

Elite professional rink hockey players (*n* = 9, age: 29.8 ± 5.77 years, weight: 79.5 ± 5.50 kg, height 180.4 ± 4.03 cm, all measurements mean ± standard deviation) participated voluntarily in the study, whereas the goalkeepers were not included. In the two seasons analysed, the team won the Spanish First Division championship and seven of the nine players of the sample played for their respective national teams in official tournaments. The data analysed came from daily player monitoring, in which player activities were routinely measured throughout the season. The experimental procedures used in this study were approved by the local Ethics and Scientific Committee and all the players signed an informed consent form before participating.

### Methodology

A retrospective observational study was carried out during the 2018–2019 and 2019–2020 competitive seasons (in the latter only until March 2020 due to COVID-19). A non-experimental descriptive method was used to identify the demands of rink hockey microcycle training sessions and matches and to evaluate the differences between days. A correlation analysis was conducted to describe the relationship between EL and IL metrics. The training load data were analysed with regard to the number of days before the match [match day (MD) minus X days] (Akenhead et al., 2016).

As it has been noted in the introduction, the microcycle it is the most important programming unit in team sports training. Tarragó et al. (2019) defined a microcycle as the time that elapses between competitive matches, and stated that its goal is to achieve the best training possible and consider the loads of the competitive matches within the dynamic of the weekly load, considering them the most important factor that affects the other sessions. The number of competition matches per week, days between matches and the physical condition of the players and the team, among other conditioning factors, affected the structure of each microcycle throughout the season. Due to all these variations, the inclusion criteria for the standard microcycles analysed were: (1) microcycles with only one match per week, (2) microcycles with four training sessions before the match, (3) microcycles with 1 day of rest after the previous match, and (4) microcycles in which the matches were official. Once the microcycles had been chosen, the inclusion criteria for the players analysed in each session were: (1) the player had to complete the entire session and (2) the player had to be available for the match. With these criteria, a total of 23 in-season microcycles were chosen out of a total of 75, with 20 MD-4 sessions, 23 MD-3 sessions, 20 MD-2 sessions, 11 MD-1 sessions, and 8 MD official matches. The mean durations (and standard deviations) of the sessions were, 62.3 ± 4.26 min for the MD-4, 85.4 ± 4.91 min for MD-3, 76.5 ± 8.46 min for MD-2, 56.1 ± 3.24 min for MD-1, and 90.4 ± 4.99 min for MD. The missing session data (for example, only 11 MD-1 sessions of the 23 microcycles were analysed) were due to factors beyond the researcher's control (e.g., technical issues with equipment) or because training sessions had been arranged on other training courts and facilities for logistical and schedule reasons. The matches and specific in-court training sessions analysed were always played on the same court (always at home), ensuring the same environmental conditions.

**Figure 1:**
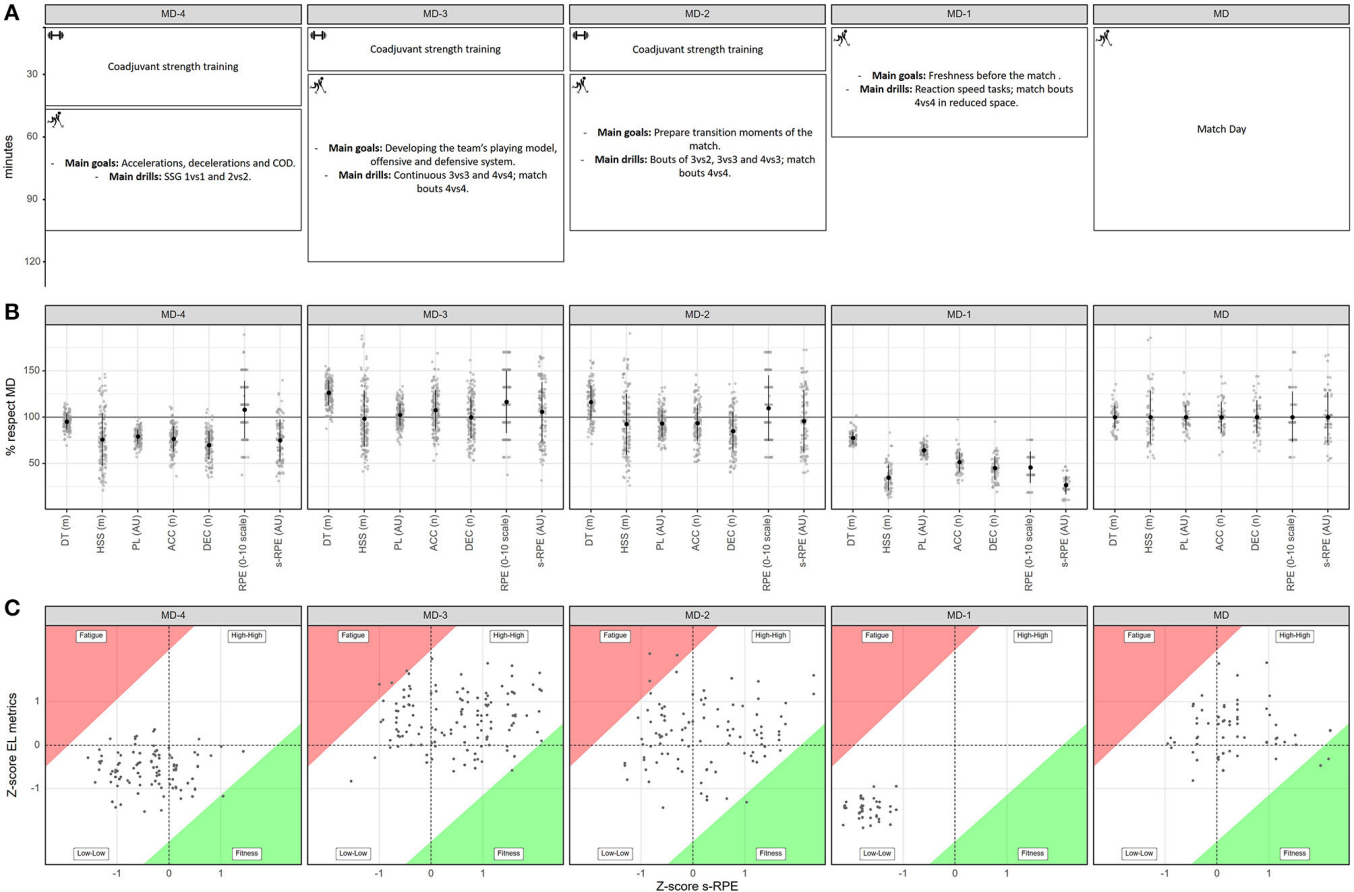
Integrative approach overview of a standard microcycle. **(A)** Main goals, characteristics, and training drills used in each session. **(B)** Mean, standard deviation and data distribution of each session expressed as a percentage of match values. **(C)** Distribution of the mean of the EL metrics *z*-scores (EL metrics *z*-scores) and the IL s-RPE *z*-scores of pooled data from all sessions. DT, Distance travelled; HSS, High-speed skating; PL, Player Load; ACC, High-intensity accelerations; DEC, High-intensity decelerations; RPE, Declared rate of perceived exertion; s-RPE, Rate of perceived exertion × total duration of session; MD, Match Day.

The training sessions of the microcyles analysed in this research were always comprised of an integrated content (i.e., tactical, technical, and physical factors were amalgamated) and in line with the structured training and structured microcycle methodology (Tarragó et al., 2019). The main goals and drills used in each training session are provided in Figure 1.

### Procedures

Data logging to evaluate EL was performed with a local positioning system (WIMU PRO™, Realtrack Systems SL) and its corresponding software (SPRO™, Realtrack Systems SL, version 962). The devices were fitted to the upper back using tight harnesses. The WIMU PRO™ features four 3D accelerometers (full-scale out output ranges are ± 16 g, ± 16 g, ± 32 g, ± 400 g, 100 Hz sample frequency), three gyroscopes (8,000°/s full-scale out output range, 100 Hz sample frequency), a 3D magnetometer (100 Hz sample frequency), a GPS (10 Hz sample frequency), and ultra-wideband (18 Hz sample frequency). The ultra-wideband system was installed on the court as follows: six antennae with ultra-wideband technology were placed 5 m away from the perimeter line of the field. The WIMU PRO system presented a high intra-class correlation coefficient value for the x-coordinate (0.65), a very high value for the y-coordinate (0.85) and a good % technical error of measurement: two (Bastida Castillo et al., 2019).

The following variables were calculated in absolute terms to describe EL: distance travelled in metres (DT; m); high-speed skating, distance covered above 18 km/h in metres (HSS >18 km·h^−1^; m); player load, vector magnitude, expressed as the square root of the sum of the squared instantaneous rates of change in acceleration in each one of the three planes divided by 100 in arbitrary units (PL; AU); number of high-intensity accelerations (ACC; >2 m·s^−2^; n) and number of high-intensity decelerations (DEC; >2 m·s^−2^; n). All of these variables were selected because they were the most commonly used ones in other competition load descriptive studies and the thresholds used were the ones emphasised most in indoor sport research and previously described in rink hockey (Fernández et al., 2020b).

Each player's declared rate of perceived exertion (declared RPE) was collected ~30 min after each specific in-court training session or match using the 0–10 point Borg's rate of perceived exertion scale modified by Foster et al. (2001), with its respective verbal anchors, in order to collect subjective IL estimations. All the players were familiar with the use of the scale. A smartphone app was used to avoid bias and maintain subjectivity in player response. Subsequently, specific in-court session declared RPE (s-RPE) was calculated by multiplying the specific session (or game) duration in minutes by the individual declared RPE scores for the training (or game) and was presented in arbitrary units (a.u.) (Foster et al., 2001). s-RPE has been proposed as a cost-effective alternative to heart rate-based methods as a global measure of training intensity that may more accurately quantify IL in intermittent sports (Impellizzeri et al., 2004; Paulson et al., 2015). Both declared RPE (0–10 point scale) and s-RPE (a.u.) results were used for further analyses.

### Statistical Analysis

All statistical analyses were conducted with RStudio version 1.3.1093 (RStudio, Inc.) and “readxls,” “tidyr,” “dplyr,” “ggridges,” “plyr,” “ggplot2,” “scales,” “viridis,” “pipeR,” “effsize,” “bootES,” “officer,” and “rvg” packages. Descriptive results were reported as mean ± standard deviation and a *z*-score analysis was conducted with the pooled s-RPE data and the mean of all *z*-score EL variable pooled data. The data failed all the tests for homogeneity of variance (Levene's test) and most of the variables and MD for normality (Shapiro–Wilk test). A bootstrap confidence interval (CI) approach was used to perform the hypothesis test to assess the differences between MDs because the assumptions of this method were aligned with our data (Tian, 2010). A residual resampling model with 10,000 bootstrap samples and 95% bias-corrected and accelerated method (BCa 95% CI) was used to calculate the CI of *F*-values of repeated-measures ANOVA for each variable and established that the null hypothesis, that there were no differences, was true if one fell within the CI limits (Plonsky, 2015). The same bootstrap CI approach with a simple resampling model was used to evaluate the *post hoc* pairwise comparisons. The mean difference and effect size value was computed and presented as pairwise change. Thresholds for effect size (ES) statistics were (ES <0.20), trivial; (0.20 < ES <0.59), small; (0.60 < ES <1.19), moderate; (1.20 < ES <1.99), large; and (ES > 2.0), very large (Hopkins et al., 2009). Finally, a bootstrapped Pearson correlation test was conducted to quantify the correlation between s-RPE and all the EL variables. The magnitude of correlation coefficients, according to Hopkins (2016), was considered trivial (*r* < 0.1), small (0.1 < *r* < 0.3), moderate (0.3 < *r* < 0.5), large (0.5 < *r* < 0.7), very large (0.7 < *r* < 0.9), almost perfect (*r* > 0.9), or perfect (*r* = 1). All the reported *P*-values were the likelihoods of the absolute effect sizes being observed if the null hypothesis of zero difference was true (Plonsky, 2015).

## Results

The EL and IL descriptive results are represented in Figure 1 and in Table 1. The MD-3 training sessions were the ones with the highest values (except the HSS variable, where the MD is the session with the highest values). In addition, Figure 1 presents an overview of all the microcycle mean values, with descriptive results relative to match values and *z*-score values of EL (presented as the mean of all *z*-score EL variables) and s-RPE metric for each training session; MD-1 sessions were the ones with the lowest relative load values and the lowest EL and IL demands measured in *z*-score values.

**Table 1:** Descriptive statistics (mean ± standard deviation) for all variables each match day.

**Variables**	**MD-4**	**MD-3**	**MD-2**	**MD-1**	**MD**
DT (m)	5,289 ± 466	7,035 ± 754	6,463 ± 1,014	4,324 ± 379	5,568 ± 750
HSS (m)	560 ± 212	727 ± 225	683 ± 245	256 ± 103	739 ± 209
PL (a.u.)	26.9 ± 2.70	34.8 ± 4.15	31.6 ± 4.85	21.8 ± 2.10	34.0 ± 4.54
ACC (*n*)	122 ± 22.0	172 ± 33.7	149 ± 31.9	82.4 ± 17.6	160 ± 26.6
DEC (*n*)	99.9 ± 21.7	143 ± 31.9	121 ± 30.1	64.4 ± 18.3	143 ± 25.8
Declared RPE (0–10 scale)	5.71 ± 1.64	6.16 ± 1.78	5.80 ± 1.87	2.43 ± 0.90	5.29 ± 1.45
s-RPE (a.u.)	356 ± 107	501 ± 153	454 ± 158	126 ± 47.3	474 ± 129

*DT, Distance travelled; HSS, High-speed skating; PL, Player Load; ACC, High-intensity accelerations; DEC, High-intensity decelerations; RPE, Rate of perceived exertion; s-RPE, Rate of perceived exertion × total duration of session; MD, Match day*.

The ANOVA results of all the variables studied are presented in Table 2, there being significant differences in all the metrics analysed. Finally, the change of each variable in each pairwise comparison between the different MD is represented in Table 3 in the form of absolute changes (in the respective units) and effect size changes (in Cohen D units). MD-3 were the training sessions most similar to the match, except in DT, ACC, and declared RPE, in which it surpassed the match values; on the other hand, MD-1 were the training sessions with the most significantly lowest values in all the metrics studied compared to the other training sessions and the match.

**Table 2:** Bootstrap ANOVA results for each variable.

**Variable**	***F***	Bootstrap*			
		**Bias**	**Std. Error**	**BCa 95%CI Lower**	**BCa 95%CI Upper**
DT (m)	242	24.1	25.7	185	266.3
HSS (m)	92.4	9.52	12.0	65.1	106.0
PL (a.u.)	229	22.9	23.7	172	251
ACC (*n*)	224	22.0	23.0	167	245
DEC (*n*)	261	25.3	26.6	199	286
Declared RPE	70.5	10.2	10.9	45.4	81.1
(0–10 scale)					
s-RPE (a.u.)	111	15.8	15.1	80.5	124

DT, Distance travelled; HSS, High-speed skating; PL, Player Load; ACC, High-intensity accelerations; DEC, High intensity-decelerations; RPE, Rate of perceived exertion; s-RPE, Rate of perceived exertion × total duration of session. The null hypothesis was rejected if 1 did not fall within BCa 95% CI limits. *Bootstrap results are based on 10,000 bootstrap samples.

**Table 3:** 95% Confidence interval of absolute change and effect size change between match days.

**Pairwise**		**DT (m)**		**HSS (m)**		**PL (a.u.)**		**ACC (*****n*****)**		**DEC (*****n*****)**		**Declared RPE (0–10 scale)**		**s-RPE (a.u.)**	
		**95CI lwr**.	**95CI upr**.	**95CI lwr**.	**95CI upr**.	**95CI lwr**.	**95CI upr**.	**95CI lwr**.	**95CI upr**.	**95CI lwr**.	**95CI upr**.	**95CI lwr**.	**95CI upr**.	**95CI lwr**.	**95CI upr**.
MD - MD-4	Abs. Δ	**74.6**	**488**	**118**	**246**	**5.93**	**8.47**	**29.9**	**45.5**	**35.9**	**50.9**	−0.91	0.09	**78.9**	**158**
	ES Δ	**0.12**	**0.85**	**0.54**	**1.14**	**1.73**	**2.49**	**1.23**	**1.93**	**1.53**	**2.21**	−0.58	0.05	**0.68**	**1.35**
MD - MD-3	Abs. Δ	**−1,687**	**−1,241**	−48.4	77.5	−2.11	0.60	**−20.5**	**−3.38**	−8.01	8.73	**−1.33**	**−0.36**	−68.0	17.1
	ES Δ	**−2.28**	**−1.57**	−0.23	0.35	−0.50	0.13	**−0.65**	**−0.10**	−0.27	0.27	**−0.82**	**−0.21**	−0.48	0.11
MD - MD-2	Abs. Δ	**−1,141**	**−634**	−6.35	127	**0.97**	**3.80**	**1.91**	**19.1**	**13.4**	**30.2**	−1.04	0.03	−25.5	65.6
	ES Δ	**−1.23**	**−0.66**	−0.04	0.52	**0.19**	**0.79**	**0.06**	**0.62**	**0.45**	**1.03**	−0.60	0.01	−0.18	0.44
MD - MD-1	Abs. Δ	**1,035**	**1,457**	**429**	**546**	**10.9**	**13.5**	**69.7**	**85.5**	**70.9**	**86.6**	**2.42**	**3.35**	**313**	**386**
	ES Δ	**1.68**	**2.66**	**2.52**	**3.53**	**2.89**	**4.14**	**2.79**	**4.11**	**2.89**	**4.10**	**1.86**	**2.68**	**2.81**	**3.86**
MD-4 - MD-3	Abs. Δ	**−1,878**	**−1,606**	**−217**	**−117**	**−8.71**	**−7.14**	**−56.2**	**−43.5**	**−49.4**	**−37.1**	−0.89	0.01	**−179**	**−111**
	ES Δ	**−3.05**	**−2.41**	**−0.98**	**−0.53**	**−2.48**	**−1.92**	**−1.96**	**−1.46**	**−1.79**	**−1.31**	−0.52	0.00	**−1.36**	**−0.81**
MD-4 - MD-2	Abs. Δ	**−1,356**	**−989**	**−175**	**−68.0**	**−5.72**	**−3.92**	**−33.5**	**−20.6**	**−27.7**	**−15.4**	−0.58	0.39	**−136**	**−61.8**
	ES Δ	**−1.75**	**−1.21**	**−0.76**	**−0.30**	**−1.45**	**−0.98**	**−1.22**	**−0.74**	**−1.06**	**−0.57**	−0.33	0.24	**−1.01**	**−0.46**
MD-4 - MD-1	Abs. Δ	**838**	**1,071**	**262**	**346**	**4.31**	**5.68**	**34.5**	**45.3**	**29.9**	**40.9**	**2.86**	**3.71**	**205**	**256**
	ES Δ	**1.79**	**2.57**	**1.41**	**1.92**	**1.64**	**2.29**	**1.54**	**2.29**	**1.36**	**2.06**	**1.86**	**2.60**	**2.10**	**2.81**
MD-3 - MD-2	Abs. Δ	**362**	**769**	−9.03	97.7	**2.12**	**4.12**	**15.3**	**29.8**	**14.6**	**28.3**	−0.13	0.83	**4.78**	**85.9**
	ES Δ	**0.41**	**0.88**	−0.04	0.42	**0.45**	**0.94**	**0.45**	**0.91**	**0.46**	**0.92**	−0.07	0.46	**0.03**	**0.57**
MD-3 - MD-1	Abs. Δ	**2,556**	**2,843**	**430**	**512**	**12.1**	**13.7**	**82.9**	**96.0**	**72.1**	**84.9**	**3.30**	**4.14**	**343**	**404**
	ES Δ	**3.61**	**4.54**	**2.13**	**2.66**	**3.14**	**3.91**	**2.64**	**3.37**	**2.42**	**3.10**	**1.97**	**2.63**	**2.45**	**3.10**
MD-2 - MD-1	Abs. Δ	**1,954**	**2,328**	**380**	**474**	**8.89**	**10.7**	**60.4**	**73.6**	**50.6**	**63.5**	**2.91**	**3.84**	**294**	**363**
	ES Δ	**2.17**	**2.85**	**1.78**	**2.32**	**2.08**	**2.66**	**2.05**	**2.74**	**1.81**	**2.45**	**1.73**	**2.34**	**2.13**	**2.72**

*The confidence intervals are based on the BCa method and 10,000 bootstrap samples. Significant differences (p < 0.05) are displayed in bold*.

*DT, Distance travelled; HSS, High-speed skating; PL, Player Load; ACC, High-intensity accelerations; DEC, High-intensity decelerations; RPE, Rate of perceived exertion; s-RPE, Rate of perceived exertion × total duration of session; MD, Match Day; CI, Confidence interval; ES, Effect Size; lwr, lower; upr, upper*.

**Figure 2:**
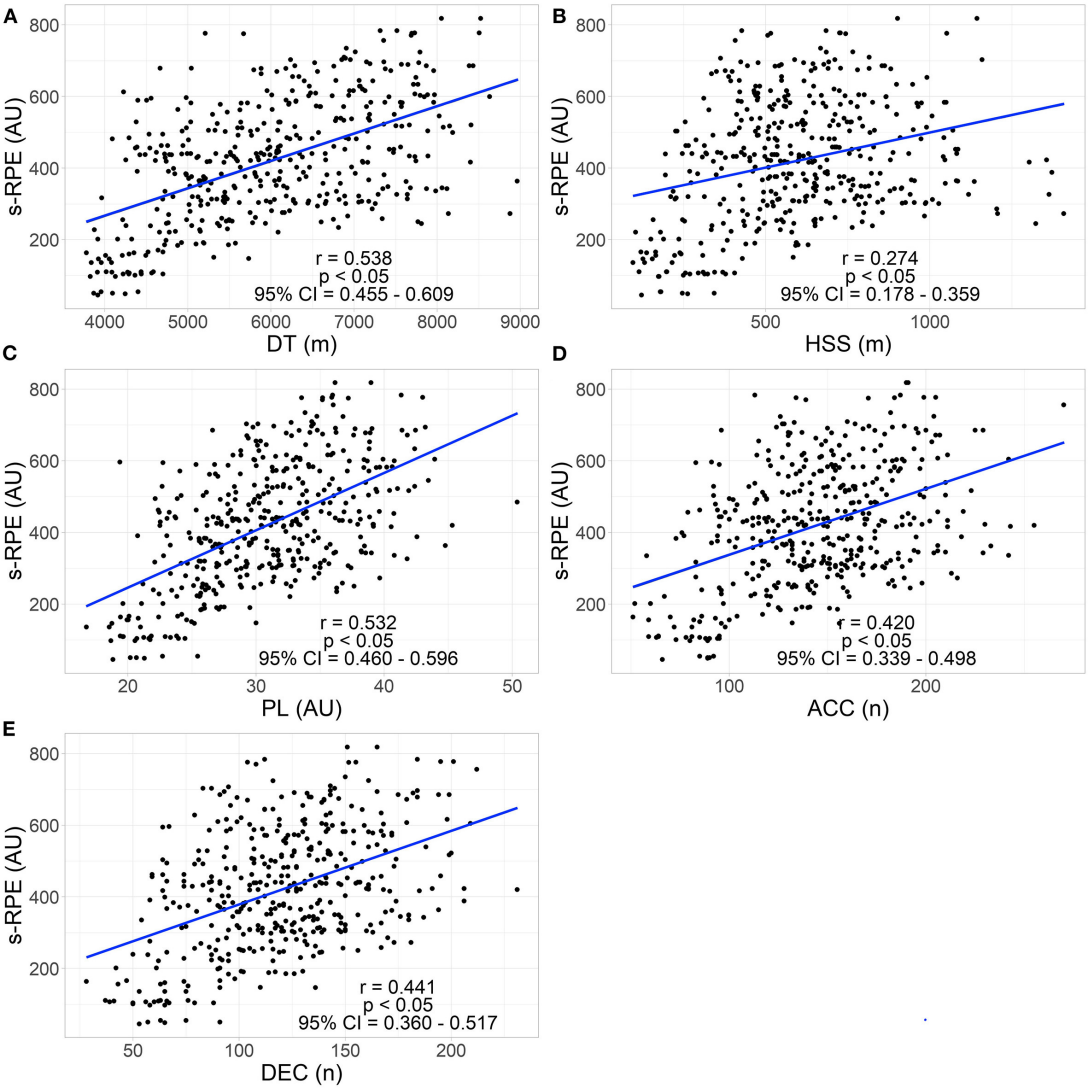
Correlation between EL metrics and session RPE (s-RPE). **(A)** s-RPE and distance travelled. **(B)** s-RPE and high-speed skating distance. **(C)** s-RPE and Player Load. **(D)** s-RPE and number of high-intensity accelerations. **(E)** s-RPE and number of high-intensity decelerations. DT, Distance travelled; HSS, High-speed skating; PL, Player Load; ACC, High-intensity accelerations; DEC, High-intensity decelerations; s-RPE, Rate of perceived exertion × total duration of session.

The correlation data analysed between EL and IL (s-RPE) are shown in Figure 2. There was a large positive correlation with DT and PL variables, moderate with ACC and DEC variables and a small positive correlation with the HSS metric.

## Discussion

The aims of this research were (i) to present an integrative approach to the EL and IL dynamics for monitoring the fitness and fatigue status of specific in-court training sessions of an elite rink hockey team for a standard microcycle, (ii) to evaluate the EL and IL differences between all the training sessions and matches, and (iii) to assess the correlation between EL and IL metrics. To the best of our knowledge, this is the first study to carefully describe the EL and IL dynamics of a microcycle of an elite rink hockey team. In this regard, our results revealed an inverted “*U*-shape” load dynamics in which MD-3 and MD-2 were the two training sessions with the greatest EL and IL of the microcycle in comparison with the other sessions and slightly above the match. Finally, and with regard to the association between EL and IL metrics, large and moderate correlations were found between volume-related variables and s-RPE and low correlations were found between high-intensity-related variables and s-RPE.

### Integrative Approach to Training Loads

One of the novelties presented by this study was the determination of the EL and IL dynamics during a standard microcycle comprised of two consecutive seasons. Figure 1 provides a visual overview of a standard microcycle content (Figure 1A), training load dynamics (Figure 1B) and an integrative approach of EL and IL for player fitness and fatigue status monitoring based on the proposals by Gabbett et al. (2017) and Delaney et al. (2018) (Figure 1C).

With regard to load dynamics, our monitoring approach showed that both EL and IL represented an inverted “*U*-shape” curve throughout a standard microcycle (Figure 1B). Regarding EL, the inverted “*U*-shape” presented is like other EL dynamics described elsewhere in other team sport microcycles, such as soccer (Akenhead et al., 2016; Los Arcos et al., 2017; Owen et al., 2017; Martín-García et al., 2018). Other research in soccer showed a decrease from MD-4 sessions to MD (Stevens et al., 2017), and finally, Malone et al. (2014), also in soccer, found no differences between MD-4, MD-3, and MD-2 training sessions. This inverted “*U*-shape” curve seems to correspond to a tapering strategy (Martín-García et al., 2018) in which EL decreases as match day approaches. Interestingly, the most notable characteristic of IL dynamics in our study was the difference between the inverted “*U*-shape” of the s-RPE metric (following the same pattern as EL metrics) versus the flat shape described by the declared RPE (0–10 point scale) variable throughout MD-4, MD-3, and MD-2 training sessions (Figure 1B). This declared RPE (0–10 point scale) dynamic might be explained by the fact that this variable is a nominal score given by the player that mainly describes mean training intensity (Haddad et al., 2017), while the s-RPE metric takes not only the intensity but also the duration of the session into consideration (Foster et al., 2001). Excepting MD-1, MD-4 is the shortest of the other three types of training days; this is why, when multiplying the declared RPE by time, the s-RPE was lowest in MD-4 training sessions.

Finally, Figure 1C presents the IL and EL metrics merged into an integrated system using pooled data *z*-scores; this type of load monitoring approach, based on Gabbett et al. (2017) and Delaney et al. (2018), provides a monitoring tool that places the players in the fitness-fatigue continuum throughout the different microcycle sessions without using tests. This approach uses the relationship between IL and EL metrics in attempt to detect uncoupling between metrics, in other words, attempt to detect when a given EL had a lower (fitness) or greater (fatigue) IL response. Moreover, the use of pooled data helps the observer to see the type of training session and its dispersion with respect to the others, and the quadrant in which it is located intra and inter sessions. In this regard, Figure 1C shows that MD-4 and MD-1 sessions tend to place, with low dispersion, the players in a “low EL and IL” zone, guaranteeing a recovery process, especially if combined with an optimal well-being status (Gabbett et al., 2017). On the contrary, in MD-3 and MD-2 sessions, as well as in MD, in which higher loads were recorded, most of the players were placed in a “high EL and IL” zone with a tendency towards dispersion towards the fitness or fatigue zones. This indicates that when players are exposed to high loads, and although most of them will exhibit an expected outcome response (“high El and IL”), the chances of having an unexpected response that would represent an improved fitness status or a fatigue process might be increased. Although this is beyond the scope of this study, such individual unexpected responses (fitness and fatigue) merit further attention in order to individualise the training process better by adjusting training loads and modifying training content.

### Differences Between Sessions in the Microcycle

The second aim of this study allowed us to compare training sessions and match EL and IL. One of the main findings was that in the MD-4 training sessions, all EL variables and s-RPE were below the match and MD-3 and MD-2 training sessions, but declared RPE was not different from the match and MD-3 and MD-2 training sessions. The differences in IL metrics (s-RPE vs. declared RPE), as previously stated, can be explained by the difference in definition of the metrics: a nominal intensity score reported by declared RPE versus the inclusion of the duration of the session in s-RPE. Moreover, this specific difference in MD-4 sessions and the rest of the sessions might also be accounted for by the fact that the players were exposed to the longest weekly (45 min) coadjuvant strength training (Gómez et al., 2019) at the gym followed by in-court sessions focusing on SSG (Figure 1A). Therefore, coadjuvant strength training in the gym just before the beginning of the specific training session could have had an impact on the declared RPE measured. This content training arrangement has been defined as concurrent training (Fyfe et al., 2014), which might have an effect on EL variables of the specific in-court part of the session (lowering the load) and an effect on the declared RPE (increasing the load), but not on the s-RPE because of the shortert in-court training time. In line with our results, Enright (2014) showed that in soccer, when resistance training was performed before specific soccer training, the specific training declared RPE was significantly higher than the inverse training sequence. Although several mechanisms may account for the difference between IL variables, none of them alone can definitely justify the results. However, knowing the reality of IL and EL dynamics can help staff members to take decisions to optimise MD-4 sessions loads, for example in this specific scenario, separating the strength session from the in-court session (into morning—afternoon sessions) would allow for a greater recovery between sessions that would mean that the perception of effort of the first session did not excessively affect the second one.

Another relevant finding regarding MD comparison was that MD-3 and MD-2 training sessions presented the greatest load demands compared to MD. MD-3 training sessions were superior in DT, ACC, and declared RPE, although no significant differences were found between HSS, PL, DEC, and s-RPE compared to MD; MD-2 sessions were significantly superior in DT; equal in HSS, declared RPE and s-RPE and significantly lower in PL, ACC, and DEC with regard to MD (Table 3). These results were similar to those reported by Illa et al. (2020) in futsal, where EL was equal to or greater than MD in the central part of the microcycle but different to outdoor team sports, such as soccer (Stevens et al., 2017; Martín-García et al., 2018), where it proved impossible to reach MD levels in the training sessions and especially in MD-2 training sessions. Unlike indoor team sports, more time may be required to recover from intense pre-match training sessions in outdoor team sports, and for this reason most of the training load was prescribed between MD-4 and MD-3 sessions. The training contents prescribed in MD-3 and MD-2 training sessions described in Figure 1A could explain the greater load demands in EL and IL (in some metrics); in line with this, some research in basketball (Vazquez Guerrero et al., 2018) suggests that the bigger the space used in specific in-court drills the greater the EL imposed upon the players. The use of roller skates in rink hockey makes it easy to move around the court and more difficult to remain motionless in one place, and this could be a reason for the higher absolute values of distance, and high-speed distance than in other indoor sports (Fernández et al., 2020b). Another interesting point is that DEC is a variable that is always lower on training days than on the match days (or at most equal). The main point is that, as mentioned in the introduction, DEC in rink hockey may be the variable that makes a difference; for example, when a player returns from an attacking phase to a defensive phase an intense and short deceleration may make the difference between a good defensive transition (timely, fast, and effective) and a bad one (late and tactically un—successful). Finally, as described in Figure 1C, all EL and IL metrics (based on the s-RPE metric) presented a major dispersion in MD-3 and MD-2 sessions, albeit with a tendency to be in the “high EL and high IL” quadrant, particularly MD-3 sessions.

Finally, with regard to MD comparison, it is also interesting to emphasise that there was a decrease in all the variables studied as match day approached (MD-1 sessions). The consistent finding of a reduction in MD-1 training sessions of EL and IL denotes a tapering strategy to guarantee freshness before competition. Many studies in team sports have demonstrated that this strategy is effective in ensuring that players will be totally competition-fit (Akenhead et al., 2016; Los Arcos et al., 2017; Owen et al., 2017; Martín-García et al., 2018; Illa et al., 2020). More specifically, if we examine competitive match loads in the weekly load dynamic, considering them as an important factor that conditions the other training sessions (Tarragó et al., 2019); then the MD-1 sessions tapering strategy in team sports consists of lowering EL and IL, ensuring recovery and guaranteeing players' match readiness (Svilar et al., 2019). In summary, and as described in Figure 1C, MD-1 presented a “low EL and low IL” profile with a low data dispersion, ensuring a consistent tapering strategy in the team analysed.

### Correlation Between EL and IL

The third and final objective of this research was to examine the correlations between EL and IL. The analysis of the relationship between the EL variables studied and the s-RPE IL metric is presented in the Figure 2. The correlations studied in the team analysed ranged from high to low in all the metrics selected; DT and PL were the two variables with the highest values of correlation with s-RPE with high values; HSS was the lowest correlated variable with s-RPE with low results. The volume metrics (DT and PL) findings are in line with those of Scanlan et al. (2014) in basketball, who found that the relationship between accelerometer training load data (similar to PL metric) and s-RPE was very similar to the correlation of our research. In contrast, field-based team sports presented stronger relationships between volume EL metrics and IL (Burgess et al., 2006; Scott et al., 2013) than indoor team sports. On the other hand, high-intensity metrics such as HSS presented the lowest correlation with IL, suggesting, on the one hand, and in line with Fernández et al. (2020b), that high-speed movement may be maintained through the use of inertia from a previous effort and not through the player's continual effort, whereby the HSS metric does not account for the players' entire internal response; and on the other hand, in line with Scott et al. (2013), that as the speed of EL criterion increases (the HSS threshold was 18 km·h^−1^), the strength of its relationship with s-RPE becomes weaker. Modern rink hockey is based on a very high pace of skating and of ball possession (Perez, 2017). In addition, professional teams seek faster offensive and defensive transitions, which probably increases the HSS volume per match as in soccer, where high—speed running distances have increased in recent years (Bush et al., 2015). This suggests the need for more research into HSS and its degree of correlation with IL and also, into absolute and relative thresholds in HSS. Finally, the results of the correlation analysed suggest that not only do EL variables affect IL response, but also that individual characteristics, psychological status, health, nutrition and the environment, among other intrinsic and extrinsic factors, could affect this response (Impellizzeri et al., 2019). Therefore, taking the different degree of association between EL and IL metrics (ranging from high-to-moderate for volume EL metrics and s-RPE and moderate-to-low for intensity EL metrics and s-RPE) into account, both variables should be measured, since they offer complementary information about conditional demands and elite rink hockey players' response to these demands, respectively.

## Conclusions

This research, the first of its kind with an elite rink hockey team, presents an integrative approach to the EL and IL of players for monitoring fitness and fatigue status during a standard microcycle. As a skating sport, DEC in rink hockey could be the variable that make a difference in match situations, possibly being this the reason why DEC was the only (along with HSS) EL variable that did not surpass the match volume during a standard microcycle. An inverted “*U*-shape” load dynamics with a load peak between MD-3 and MD-2 sessions, a mismatch between declared RPE and s-RPE metric in MD-4 sessions and a high correlation between volume EL variables and IL (s-RPE) were the most important findings in this team's microcycle. This could provide team sport staff members (coaches, strength and conditioning coaches, and physicians) with a deeper understanding of load distribution throughout the microcycle. The use of *z*-scores analyses integrating EL and IL builds up an objective decision-support tool for changing and adapting training loads and content. This study is based on a single elite rink hockey team with international top-level players, and the findings provide a basis for the quantification of a standard microcycle, constituting a point of departure for other research into EL and IL in this sport as well as in other team sports.

## Data Availability Statement

The raw data supporting the conclusions of this article will be made available by the authors, without undue reservation.

## Ethics Statement

The studies involving human participants were reviewed and approved by the ethics committee for clinical research of the Catalan Sports Council 29/CEICGC/2020. The patients/participants provided their written informed consent to participate in this study.

## Author Contributions

DF participated in the design of the study, contributed to data collection, reduction, analysis, interpretation of results, and contributed to the manuscript writing. DM participated in the interpretation of results and contributed to the manuscript writing. JC contributed to the manuscript writing. GC participated in the design of the study, contributed to interpretation of results, and manuscript writing. All authors have read and approved the final version of the manuscript and agree with the order of presentation of the authors.

## Conflict of Interest

The authors declare that the research was conducted in the absence of any commercial or financial relationships that could be construed as a potential conflict of interest.

## Abbreviations

EL: external load
IL: internal load
declared RPE: declared rate of perceived exertion
s-RPE: session rate of perceived exertion
MD: match day
MD-x: match day [MD] minus X days
DT: distance travelled
HSS: high-speed skating
ACC: high-intensity accelerations
DEC: high-intensity decelerations
ES: effect size.
